# Fronto-temporal functional disconnection precedes hippocampal atrophy in clinically confirmed multi-domain amnestic Mild Cognitive Impairment

**DOI:** 10.17179/excli2021-4191

**Published:** 2021-09-27

**Authors:** Kathryn M. Broadhouse, Natalie J. Winks, Mathew J. Summers

**Affiliations:** 1 The University of the Sunshine Coast, School of Science and Engineering, Sunshine Coast, QLD, Australia; 2 Sunshine Coast University Hospital, Sunshine Coast Hospital and Health Service, Birtinya, QLD, Australia; 3 The University of the Sunshine Coast, School of Health and Behavioural Sciences, Maroochydore, QLD, Australia

**Keywords:** mild cognitive impairment, functional magnetic resonance imaging, cognitive function, Alzheimer's Dementia, cognitive decline

## Abstract

Mild Cognitive Impairment (MCI) is fraught with high false positive diagnostic errors. The high rate of false positive diagnosis hampers attempts to identify reliable and valid biomarkers for MCI. Recent research suggests that aberrant functional neurocircuitries emerge prior to significant cognitive deficits. The aim of the present study was to examine this in clinically confirmed multi-domain amnestic-MCI (mdaMCI) using an established, multi-time point, methodology for minimizing false positive diagnosis. Structural and resting-state functional MRI data were acquired in healthy controls (HC, n=24), clinically-confirmed multi-domain amnestic-MCI (mdaMCI, n=14) and mild Alzheimer's Dementia (mAD, n=6). Group differences in cortical thickness, hippocampal volume and functional connectivity were investigated. Hippocampal subvolumes differentiated mAD from HC and mdaMCI. Functional decoupling of fronto-temporal networks implicated in memory and executive function differentiated HC and mdaMCI. Decreased functional connectivity in these networks was associated with poorer cognitive performance scores. Preliminary findings suggest the large-scale decoupling of fronto-temporal networks associated with cognitive decline precedes measurable structural neurodegeneration in clinically confirmed MCI and may represent a potential biomarker for disease progression.

## Introduction

The clinical diagnosis of dementia is preceded by an extended preclinical period of neurodegeneration without clinical symptoms, suggesting that a cascade of dynamic biomarkers precede clinical onset (Sperling et al., 2011[37]). This has led to a field-wide focused attempt to identify potential biomarkers of preclinical Alzheimer's Dementia (AD) with Mild Cognitive Impairment (MCI) as an important transitional state between healthy aging and dementia (Albert et al., 2011[1]; Petersen et al., 1999[31]; Winblad et al., 2004[46]). Currently, MCI is considered to be the earliest phase of cognitive decline preceding clinical diagnosis of dementia that can be detected by conventional clinical assessments and is therefore an important diagnostic entity in both clinical and research settings.

In the search for potential biomarkers of preclinical AD, accurate identification of individuals with MCI as a precursor to AD is of paramount importance, so that interventions can be delivered at a time when treatment efficacy is likely to be maximal. An ongoing problem in MCI research is the high rate of false positive MCI diagnosis when a single assessment point is used, with multiple longitudinal studies reporting that 28-56 % of those diagnosed with MCI are found to recover to unimpaired levels of functioning (Gauthier and Touchon, 2005[17]; Klekociuk et al., 2014[22]; Klekociuk and Summers, 2014[23]; Palmer et al., 2003[29]; Summers and Saunders, 2012[39]). There is a real potential that biomarker studies of preclinical AD studying MCI cases may be confounded by high levels of sampling heterogeneity, with a significant proportion of MCI cases identified being false-positive cases. Research exploring ways to reduce the false-positive MCI detection rate has identified multiple-domain aMCI (mdaMCI) to be a superior predictor of risk for conversion to AD than other MCI subtypes (Klekociuk and Summers, 2014[23]; Summers and Saunders, 2012[39]). The finding that mdaMCI variants have the highest reliability of diagnosis has been confirmed in multiple other studies (Belleville et al., 2014[4]; Bondi et al., 2014[5]; Clark et al., 2013[9]; Edmonds et al., 2015[13], 2016[14]; Weissberger et al., 2017[44]). Further, there is increasing evidence that repeated testing may be necessary to confirm the temporal stability of subclinical cognitive impairments required for a diagnosis of MCI and to eliminate false positive diagnostic errors (Klekociuk and Summers, 2014[23]; Summers and Saunders, 2012[39]).

Structural and functional imaging of MCI and early stages of AD have identified patterns of neurodegeneration that match the clinical symptoms and cognitive changes noted for both MCI and AD. MCI and the early stages of AD are characterized by increased neurodegeneration of the hippocampus and entorhinal cortex (Broadhouse et al., 2019[7]; Frisoni et al., 2010[16]; Mu and Gage, 2011[28]), consistent with deficits to memory and severity of cognitive impairment (Jack et al., 2000[20]; Morra et al., 2009[27]) as well as increased risk of conversion from MCI to AD (Apostolova et al., 2006[3]; Costafreda et al., 2011[10]). More recently resting-state functional MRI (rsfMRI) studies report that dysfunction in networks implicated in memory consolidation and episodic memory are associated with disease progression from MCI to AD (Liu et al., 2014[25]).

In addition to memory deficits, there is emerging evidence suggesting that a deterioration in executive functions in MCI may precede the emergence of episodic memory deficits in early stages of AD (Brandt et al., 2009[6]; Saunders and Summers, 2010[34], 2011[35]; Summers and Saunders, 2012[39]). Functional networks within the limbic system, frontal and temporal cortex have been implicated in higher order executive functioning, impulse control, cognitive flexibility and decision making (Mars et al., 2012[26]; Vincent et al., 2008[41]). It has been suggested that the functional disruptions within these neurocircuitries lead to maladaptive responses during cognitive and emotional processing, which may underpin cognitive decline in this cohort (Chen et al., 2016[8]). However, research to date examining differences in network connectivity between MCI and health controls provide mixed and somewhat conflicting results, with some studies reporting decreased connectivity (Alderson et al., 2017[2]; Dunn et al., 2014[12]; Li et al., 2017[24]; Sheng et al., 2017[36]) and others reporting increased “compensatory” connectivity (Farrar et al., 2018[15]; Sullivan et al., 2019[38]). Despite these inconsistencies, it is likely that changes to network connectivity precede volumetric loss of brain tissue, indicating that rsfMRI techniques may be capable of detecting preclinical changes in neural networks before clinical symptoms of decline to memory and executive function decline become evident.

In the present study we examine structural and rsfMRI differences between older adults with longitudinally confirmed mdaMCI, clinically diagnosed mild Alzheimer's Dementia (mAD) and age-matched healthy comparators. With this study we aim 1: to investigate the cortical and subcortical atrophy patterns and corresponding resting-state functional network signatures that distinguish HC from mdaMCI, and examine if similar changes are also evident in mAD. 2: to examine the association between neural network dysfunction and memory and executive deficits in mdaMCI. 

## Methods

### Trial design and participants

Participants aged 65 years and older were recruited from the local community by referral from health care providers as well as volunteers from community-based groups for older adults. Recruitment and selection of participants is described in the supplementary information (**Supplementary File 1)**. A total of 128 potential participants underwent telephone screening and 48 met eligibility criteria. Participants then underwent formal baseline neuropsychological assessment of cognitive function, psychological status, and health status to determine eligibility for one of 3 groups: (1) confirmed cognitively healthy older adults (HC); (2) possible multi-domain amnestic-MCI (mdaMCI), or (3) clinically confirmed mild AD (mAD). Participants in the mAD group were recruited on the basis of a pre-existing diagnosis made by a geriatrician within 2 years, with the baseline neuropsychological assessment confirming the independent diagnosis. Criteria for mdaMCI was the presence of a subclinical impairment (>1.28SD below age-referent normative means) (Klekociuk and Summers, 2014[23], Summers and Saunders, 2012[39]) on >1 test of function within a single cognitive domain (e.g. memory) with evidence of subclinical impairment across 2 or more cognitive domains at both assessments. Participants characterized as possible MCI following baseline assessment were classified as “possible mdaMCI” pending confirmation at repeat assessment. All possible mdaMCI cases underwent repeat full neuropsychological assessment 1 month later to confirm mdaMCI diagnosis. Possible mdaMCI participants who met the criteria for mdaMCI at both assessments were finally classified as clinically-confirmed mdaMCI, possible mdaMCI participants not meeting the mdaMCI criteria at the second assessment were finally classified as healthy controls. All assessments were carried out by the same registered Clinical Neuropsychologist with over 20 years of experience (MS). Following baseline and 1-month reassessment, 47 participants met eligibility criteria, as either HC (n=26), confirmed mdaMCI (n=16) or mAD (n=6) and underwent a neuro MRI scan within 1 week of final cognitive assessment. For 3 participants (1 HC, 2 mdaMCI) MRI scan was incomplete, with the final sample available for analysis being HC = 24, mdaMCI = 14, and AD = 6. 

### Ethical approval

Ethics approval was obtained through the University of the Sunshine Coast Human Research Ethics Committee (A181181) in accordance with the National Health and Medical Research Council (NHMRC) of Australia national statement on conduct in human research. All participants provided fully informed consent for participation in this study and all methods were carried out in accordance with NHMRC approved guidelines and regulations.

### Neuropsychological assessment

The assessment battery used in this study (Table 1(Tab. 1)) is derived from our prior prospective longitudinal research in MCI and healthy older adults (Klekociuk and Summers, 2014[23]; Summers and Saunders, 2012[39]) and incorporates tests that we have shown to reliably identify longitudinally confirmed cases of MCI with 85 % accuracy at a single assessment (Klekociuk and Summers, 2014[23]; Summers and Saunders, 2012[39]).

### Magnetic Resonance Imaging 

Neuro MRI scans were acquired on a 3-Tesla Siemens Skyra MRI (Germany, Erlangen) with a 64-channel head and neck receive coil. High-resolution, whole-brain, anatomical scans were acquired using a 3D T1-weighted Magnetization-Prepared Rapid-Acquisition Gradient Echo sequence (MPRAGE; TR=2200 ms, TE=1.76 ms, TI=850 ms, FOV=240 mm, 256x256 matrix, spatial resolution=0.9 mm isotropic). rsfMRI connectivity was analyzed from multi-slice, T2* echo-planar BOLD sequences acquired with eyes closed (FOV=240x240 mm, matrix size=80x80, 56 slices, slice thickness=3 mm, TR/TE=1400/30 ms, 404 volumes, multi-slice factor=4, acceleration factor=2, scan duration=9 minutes). 

### MRI processing and analysis

Processing and analysis of MRI data was undertaken by an experienced investigator (KB) who was blinded to the participant group.

*Structural analysis*: MPRAGE datasets were automatically processed to obtain reliable cortical reconstruction and volumetric segmentations using the *whole-brain* imaging analysis stream in FreeSurfer v6.0. All outputs were visually inspected and where necessary, manual edits were carried out following the FreeSurfer manual editing pipeline. Segmentation of the left and right hippocampi into the head and body subregions were performed following the *hippocampal subfield and nuclei of the amygdala* processing stream FreeSurfer v6.0 (HBT output) using the Freesurfer image analysis suite, which is documented and freely available for download online (http://surfer.nmr.mgh.harvard.edu/). All hippocampal volumes were normalized to individual total intracranial volume (the eTIV FreeSurfer output volume).

*Functional connectivity analysis*: Structural FreeSurfer processed and rsfMRI datasets were pre-processed and analyzed with the open source Matlab/SPM-based CONN toolbox (Version 16 with SPM12, conn-toolbox.org) following the default DARTEL pre-processing stream (Whitfield-Gabrieli and Nieto-Castanon, 2012[45]). Data was manually entered into CONN's GUI which enabled setup, preprocessing, denoising, first-level and second-level analysis. As with most Siemens scanners, the dummy scans are acquired as part of the prescan, along with the reference data for the multiband reconstruction to allow for magnetization stabilization to steady state. Therefore, all 404 acquired volumes were included in the CONN analysis pipeline, which is fully documented in the software documentation (Whitfield-Gabrieli and Nieto-Castanon, 2012[45]). The step-by-step processing includes: (i) *Setup and preprocessing*. Realignment of the rsfMRI images, each volume was realigned to the first volume to correct for any residual head movement. The subjects individual FreeSurfer-generated anatomical files (T1.mgz files) and gray/white and CSF masks were then imported into CONN and co-registered to the first volume of the rsfMRI data after realignment, using a 6 degrees-of-freedom linear transformation without resampling. The co-registered structural images were then standardized to the Montreal Neurological Institute (MNI) space. The transformation matrix was then applied to the individuals realigned rsfMRI images, and the gray/white and CSF masks. Finally, spatial smoothing was carried out FWHM = 8 mm). 

Slice-timing was omitted as the multi-slice EPI sequence provided sub 2000 TR). (ii) *Denoising*. Next CONN's default denoising pipeline was performed (https://web.conn-toolbox.org/fmri-methods/denoising-pipeline). Control of residual physiological and motion artefacts was performed. The head movement time-series, white matter and CSF signal were regressed out from each voxel using the aCompCor strategy (full details given in the software documentation conn-toolbox.org). Band-pass filtering of 0.01-0.08 Hz was then carried out to focus on slow-frequency fluctuations while minimizing the influence of physiological, head motion and other sources of noise. Finally, denoising outputs were evaluated from the distribution of functional connectivity values between randomly selected pairs of points within the brain within CONNs quality control plots. After denoising function connectivity plots were approximately centered distributions. (iii) *First-level analysis*. ROI-to-ROI analyses were then carried out in the CONN GUI and individual functional connectivity matrices, representing the Fisher-transformed bivariate correlation coefficient (z-scores) between each pair of ROI BOLD time-series, were then determined using the default FSL Harvard-Oxford Atlas as the ROI-to-ROI template. (iv) *Second-level analysis* carried out in the CONN GUI is described below (*Resting-state network analysis*).

### Statistical analysis

*Demographic analysis*: A Chi-squared test was used to determine significant difference in sex, living status and right-handedness sample distribution between the three diagnoses groups. A One-Way ANOVA with post-hoc Bonferroni multiple comparisons analysis was used to determine significant difference in general demographic data and psychological assessment scores between groups. Levene's test was used to assess homogeneity of variance and visual inspection of the box plot distributions for each variable within each group was conducted to assess for normality of distribution and presence of outliers. Although there was no significant difference in age between the groups, there is a significant correlation between age and brain structure and function (Reuter et al., 2012[33]; Tsvetanov et al., 2015[40]). Cognitive test scores were transformed to age-corrected scores (z-scores or scaled scores) against established norms, thereby removing any effect of age on individual test performances. Due to the potential for age to influence measure of brain structure, age was included as a covariate in the subsequent analyses of MRI measures.

*Hippocampal volume analysis*: Significant difference in left and right whole, head and body hippocampal volume segmentations were assessed with a One-Way ANCOVA. Post-hoc Bonferroni multiple comparisons analysis was used to determine group level significance. All the above statistical analyses were carried out in SPSS (IBM, Armonk, NY, USA. Release 24) and results presented are corrected p-values. 

*FreeSurfer general linear modelling*: Vertex-wise surfaced-based group analysis to determine significant difference in cortical thickness between the groups were carried out in FreeSurfer's general linear model analysis suite (Reuter et al., 2010[32]). A One-Way ANCOVA model was constructed testing the difference in cortical thickness between HC and mdaMCI and the sub-analyses of mdaMCI and mAD and HC and mAD. Cluster-wise correction for multiple comparisons was then run with a vertex-wise/cluster-forming threshold of p<0.0001 and adjusting for both hemispheres (Hagler et al., 2006[19]).

*Resting-state network analysis*: A One-Way ANCOVA analysis was carried out to investigate differences in (i) whole brain ROI-to-ROI functional connectivity (excluding brain stem and cerebellar), and (ii) a focused analysis of functional connectivity within 56 fronto-temporal-limbic ROIs (see Supplementary information, Supplementary File 2) between HC and mdaMCI and mdaMCI and mAD. Finally, a correlation analysis was constructed to investigate the association between the six memory and executive domain performance scores defined above and fronto-temporal-limbic functional connectivity across the entire participant sample. Performance scores were normalized to z-score or age standardized score and therefore age was not included as a covariate in this correlation analysis. All functional network-based statistical analyses were carried out in CONN (Whitfield-Gabrieli and Nieto-Castanon, 2012[45]) and all p-values were FDR seed level corrected.

### Power and effect size

Obtained power and effect size for parametric analyses are reported in Table 2(Tab. 2). As is evident from these analyses, significant differences were detected where very large effect sizes were present (*f* > .387) indicating that the analyses were sufficiently powered to detect meaningful differences between group performances.

## Results

### Cohort demographics

Of the 48 participants recruited 44 (female = 30, age: M 73.3, SD 5.9 yrs) had MPRAGE and rsfMRI datasets for processing. Of the 18 participants diagnosed as possible mdaMCI at baseline 16 had clinically-confirmed mdaMCI at 1-month reassessment indicating a false positive diagnosis of 11 % with the single assessment approach. There were no significant differences in handedness, sex or living status, age or years of education between groups. Full results are given in Table 2(Tab. 2).

### Neuropsychological assessments

One-Way ANOVA analyses revealed significant differences across all measures of cognitive function except for number of errors made on a simple motor test of sensorimotor function (MOT). Post-hoc analysis indicated that measures of visual episodic memory (PAL), verbal working memory (Letter Number Sequencing), visual working memory (SWM), and semantic memory/language (BNT) significantly differentiated between HC, mdaMCI and mAD. Additionally, both the AD and mdaMCI group displayed significant impairments compared to the HC group on measures of verbal episodic memory (RAVLT), motor speed (MOT), and complex sustained attention (RVP A'). Full results are given in Table 3(Tab. 3).

### Cortical thickness and hippocampal segmentation

**Figure 1:**
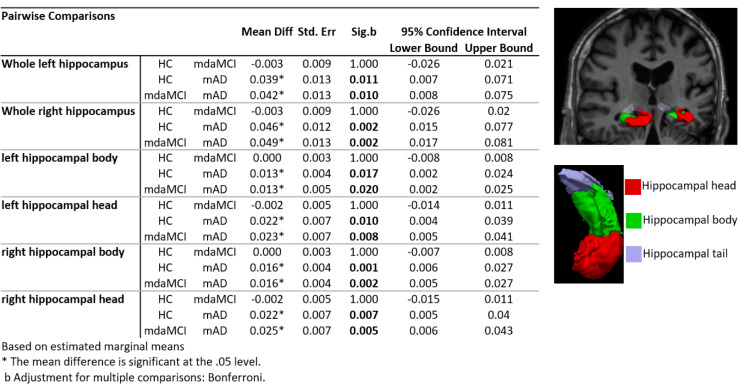
Hippocampal differentiation between the groups. Pairwise analysis results for both left and right normalized whole, head and body hippocampal segmentations. Results show significant reduction in all hippocampi segmentation volumes between mAD and mdaMCI and HC. An example of a 3D surface rendering of the left and right hippocampi volumes superimposed over the structural dataset (top right) and individual head, body and tail segmentations (bottom right) are given for reference. All hippocampal volumes were normalized to individual total intracranial volume.

There was a significant difference in both left and right whole, head and body hippocampal segmentations between mAD and both HC and mdaMCI. However, hippocampal segmentation volumes did not differentiate between HC and mdaMCI groups. Full results are provided in Figure 1(Fig. 1). Uncorrected vertex-wise surface analysis revealed very little difference in whole brain cortical thickness between HC and mdaMCI. A significant pattern of cortical thinning in the frontal and temporal lobes was observed when comparing HC versus mAD and mdaMCI versus mAD. However, this did not survive multiple comparison thresholding. Raw, uncorrected significance maps are shown in the Supplementary information (**Supplementary File 3)**.

### Functional disconnect marks early disease state

**Figure 2:**
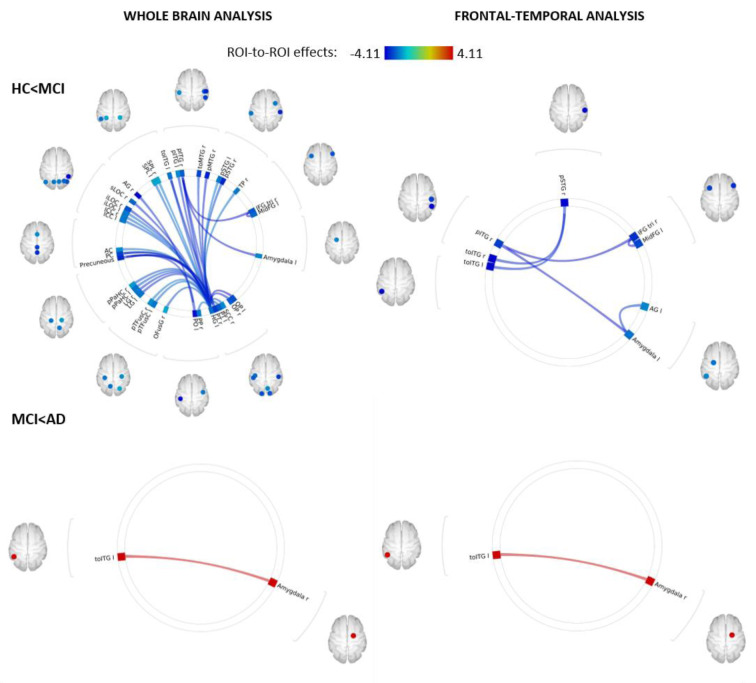
Functional decoupling with disease progression. Whole-brain ROI-to-ROI analysis carried out in CONN (top left) revealed a global decoupling in the mdaMCI group with significant reductions within and between temporal, frontal, limbic and occipital regions when compared to HC. A focused analysis including 56 pre-identified frontal, temporal and limbic regions (top right) revealed significant reductions in functional connectivity between the temporal gyrus, amygdala and frontal regions, regions implicated in higher order cognitive function. Decoupling between these regions may underpin deficits in cognitive function seen in this cohort. This decoupling pattern did not continue with disease progression. Whole-brain (bottom left) and frontal-temporal (bottom right) group comparisons between mdaMCI and mAD revealed a significant increase in functional connectivity between the right amygdala and left inferior temporal gyrus. Significant FDR corrected connectivity changes are shown between regions, with color indicating the Fisher-transformed bivariate correlation coefficient (z-scores) between each pair of ROI BOLD time-series. Figure 2 was produced using CONN secondary level analysis graphic user interface (Version 16 with SPM12, conn-toolbox.org) by author KB.

Whole-brain ROI-to-ROI analysis revealed a significant, global decoupling of the frontal-temporal and temporal-occipital regions in mdaMCI compared to HC. The focused, frontal-temporal ROI analysis revealed significant reductions in resting-state functional connectivity between the temporal gyrus, amygdala and frontal regions. Preliminary whole-brain and frontal-temporal group comparisons between mdaMCI and mAD indicated very little difference in resting-state functional connectivity; with only a significant increase in functional connectivity between the right amygdala and left inferior temporal gyrus in the mAD group. Functional connectivity group analyses are shown in Figure 2(Fig. 2). 

### Fronto-temporal functional disconnect is associated with cognitive decline

**Figure 3:**
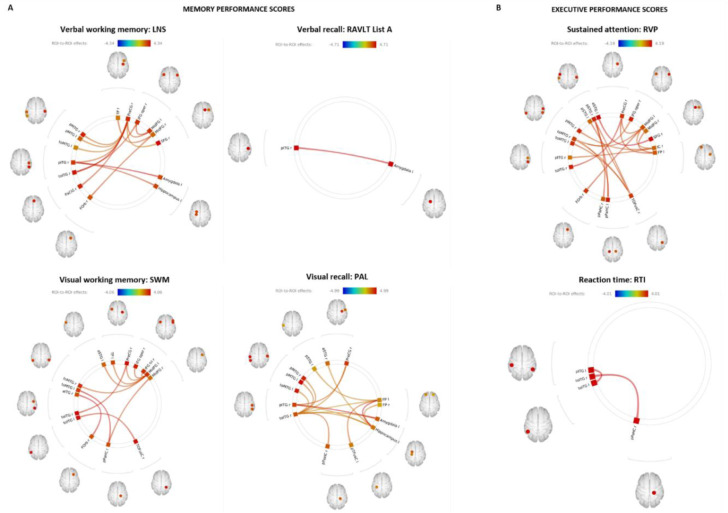
Functional connectivity correlates of memory and executive function performance scores. Bivariate correlation analysis carried out in CONN revealed significant association between increased fronto-temporal-limbic functional connectivity and improvements in (A) memory domain scores of verbal working memory (top left), verbal recall (top middle), visual working memory (bottom left) and visual recall (bottom middle). (B) executive domain analysis revealed significant association between numerous fronto-temporal-limbic networks and sustained attention performance (top right) and reaction time (bottom right). Significant FDR corrected connectivity changes are shown between regions, with color indicating the Fisher-transformed bivariate correlation coefficient (z-scores) between each pair of ROI BOLD time-series. Figure 3 was produced using CONN secondary level analysis graphic user interface (Version 16 with SPM12, conn-toolbox.org) by author KB.

Bivariate correlation analysis revealed significant association between verbal and visual working memory, visual new learning and sustained attention performance scores and fronto-temporal-limbic networks. Higher verbal recall (RAVLT list A) and reaction time (RTI-RT) performance *z-scores* were significantly correlated with increased temporal-limbic functional connectivity. Functional connectivity correlates of these cognitive domain scores are given in Figure 3(Fig. 3).

## Discussion

The use of unrefined MCI diagnostic criteria has resulted in heterogenous MCI samples being included in many studies searching for reliable biomarkers for disease and disease progression. To address this issue, in the present study we identified a longitudinally confirmed homogenous mdaMCI cohort showing that functional decoupling of fronto-temporal networks occurs prior to measurable medial temporal lobe (MTL) atrophy. Moreover, we show that dysfunction or decreased functional connectivity in these networks is associated with decreased performance on measures of episodic memory and executive function in mdaMCI. These findings concur with our previous clinical work demonstrating that deficits to attention and working memory precede episodic memory deficits in a longitudinal prospective study of MCI (Saunders and Summers, 2010[34]).

Large-scale ADNI (Alzheimer's Disease Neuroimaging Initiative) studies have highlighted that using refined MCI diagnostic criteria to improve the selection of 'true positive' MCI cases and removal of 'false positive' MCI cases may yield gains in biomarker findings (Bondi et al., 2014[5]; Edmonds et al., 2015[13]). These studies indicate that MCI studies using conventional diagnostic criteria (single timepoint assessment, lack of MCI subtype specificity) may be diluting important biomarker associations. Our preliminary results support this: even when focusing on mdaMCI, the subtype with the highest conversion rate to AD, the longitudinally confirmed mdaMCI approach identified that 11 % of the single timepoint assessed cases were false positives. Although this preliminary study has a small sample size, this rigorous diagnostic approach provides a stable, homogenous cohort and offers an opportunity to investigate potential biomarker-cognitive deficit relationships with disease progression. Adoption of this diagnostic approach by the wider research community presents an opportunity to gain much needed insight into the cascade of pathophysiological processes of AD and inform targeted intervention. 

Previous studies have shown that increased neurodegeneration of the hippocampus and entorhinal cortex coincides with memory deficits that characterize MCI, early stages of AD and conversion between the two (Frisoni et al., 2010[16]; Mu and Gage, 2011[28]). Here we show that although progressive cognitive deficits in memory and executive function discriminate mdaMCI from HC and mAD from mdaMCI, group comparison of these AD-sensitive brain regions (hippocampal subvolume and MTL atrophy patterns) lacked the same capacity to differentiate groups. This supports the cascade model, indicating that while early measurable atrophy signatures may predict conversion from MCI to AD, and HC to MCI in much larger samples (Reuter et al., 2012[33]), there may be more appropriate markers for predicting conversion from health prior to cognitive decline. Identification of these more sensitive, upstream markers in this difficult-to-diagnose cohort is key to establishing prevention strategies and understanding if it is possible to modify the course of underlying neurodegeneration. 

Resting-state fMRI allows quantification of spontaneous brain activation at rest, providing insight into the brain's functional organization in both health and disease. Recent studies focusing on aberrant functional connectivity as a potential candidate for markers of early decline have shown that the loss of long distant functional connections between lobes and hemispheres mark disease progression from HC to broadly diagnosed MCI to AD (Liu et al., 2014[25]). Network analysis has also highlighted the role of the default mode (DMN) and frontoparietal networks in MCI (Alderson et al., 2017[2]; Dunn et al., 2014[12]; Li et al., 2017[24]; Weiler et al., 2014[43]). These whole-brain network analysis approaches are emerging as a promising approach of broad MCI classification (Chen et al., 2016[8]; Wee et al., 2016[42]) with regions of the brain becoming more “functionally isolated” as cognitive decline advances. Our results in this longitudinally confirmed mdaMCI cohort build on this literature, with functional connectivity analysis showing significant decoupling within and between fronto-temporal networks and important hubs within the DMN (the posterior cingulate) in mdaMCI when compared to healthy seniors. These preliminary results signifying large scale functional decoupling in this small stable MCI subtype sample is promising in the pursuit to identify more specific markers of early decline. Particularly when considering the previously mentioned lack of specificity in cortical thickness and volume measures in the same regions. 

Functional connectivity was also shown to be significantly associated with memory and executive performance scores within the whole group. Otherwise stated, fronto-temporal functional disconnect is associated with cognitive deficits that characterize disease progression. These and the above results suggest that functional decoupling of key neurocircuitries may precede structural atrophy in the dynamic biomarker cascade. These results raise important questions around the directional relationship between functional and structural neurodegeneration. Furthermore, the lack of significant global differences in functional connectivity between mdaMCI and mAD may further support the notion that functional disconnect precedes structural atrophy in the hypothesized AD cascade theory, whereby neuronal dysfunction evolves prior to neurodegeneration. Future large-scale longitudinal studies mapping both atrophy and network disconnect coupled with longitudinally diagnosed MCI will determine whether neuronal dysfunction is the dominant pathological process prior to, or simply coincides with clinical symptom onset, thereby defining its potential as a biomarker for targeted intervention.

### Limitations

Although the above findings are encouraging, they are reported from a small sample and caution needs to be exercised when interpreting these results. Firstly, due to this small sample size group analysis between HC and MCI, the primary focus of this study, may be driven by larger numbers of healthy controls. Although there was no significant difference in demographic measures between groups, future studies with larger, age-matched cohorts will provide more reliable investigations of the neuronal dysfunction that classifies these two pathophysiologically distinct cohorts. 

Secondly, we present a cross sectional study indicating that functional connectivity is a more sensitive measure at differentiating early stage cognitive impairment than structural cortical and subcortical measures. However, the lack of follow-up data and small cohort of mAD participants means that we are unable to conclude whether dysfunction of specific networks or neuronal signatures can be utilized to predict conversion from HC to MCI to AD. Future longitudinal larger-scale studies will provide insight into the distinct neuronal underpinnings that characterize these clinical stages of disease, but also identify the functional signature that predicts or precedes progression. 

Finally, we have only examined multi-domain amnestic MCI. The clinical phenotypes of amnestic and non-amnestic MCI are believed to be underpinned by different underlying pathophysiologies and disease trajectories. Although aMCI displays a higher rate of risk of conversion to AD than naMCI (Gauthier et al., 2006[17]; Petersen et al., 2009[30]), the disease trajectory for naMCI is less well defined (Jungwirth et al., 2012[21]) , but appears to be linked to higher risk and more frequent conversion to other dementia types (e.g. vascular dementia) compared to those whom are cognitively intact (Csukly et al., 2016[11]). Network analysis of both subtypes may provide insight into the underlying aberrant neurocircuitries that define subtype and disease trajectory as well as aid in classification.

In summary, our preliminary findings suggest that large-scale decoupling of fronto-temporal networks differentiate mdaMCI from healthy controls, that this disconnect is associated with cognitive decline and that this neuronal dysfunction precedes measurable structural neurodegeneration between these two cohorts. Further longitudinal studies investigating the neuronal underpinnings of disease progression will provide insight into potential functional biomarkers and establish the significance of functional decoupling within the sequential model of dynamic biomarkers of the Alzheimer's pathological cascade. 

## Disclosure statement

The authors report no potential conflicts of interest to disclose.

## Author contribution statement

KB – design and conceptualization of the study; data analysis; data interpretation; manuscript preparation

NW – data collection/acquisition, revision of manuscript

MS – design and conceptualization of the study; data analysis; data interpretation; data acquisition; participant recruitment and assessment; manuscript preparation and revision.

## Data availability

The data that support the findings of this study are available from the corresponding author, MS, upon reasonable request.

## Acknowledgements

The authors have no conflicts of interest to declare. There are no funding sources to disclose. The authors thank the Sunshine Coast Mind and Neuroscience – Thompson Institute of the University of the Sunshine Coast for use of the magnetic resonance imaging scanner used in this study.

## Supplementary Material

Supplementary information

## Figures and Tables

**Table 1:**
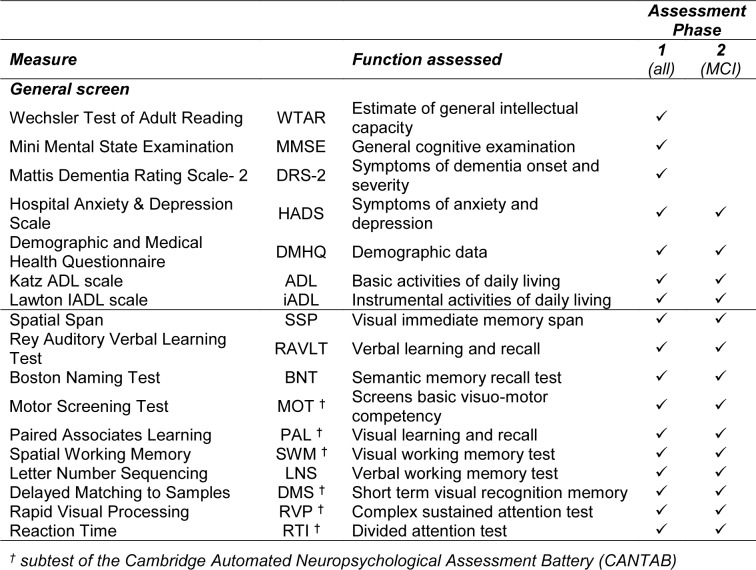
Neuropsychological assessment battery

**Table 2:**
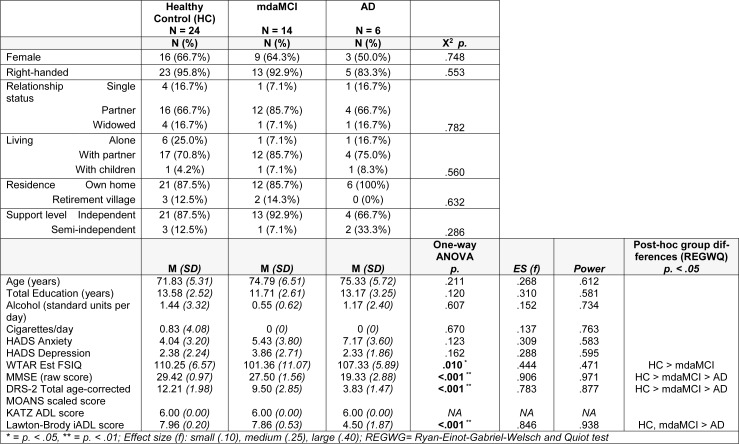
Demographic and screening measures for healthy control, multi-domain amnestic MCI, and AD participants

**Table 3:**
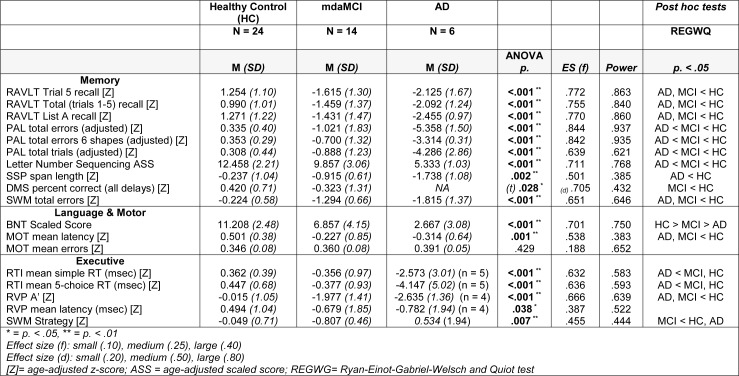
Neuropsychological measures for healthy control, multi-domain amnestic MCI, and AD participants
