# Supplementary material for: Fronto-temporal functional disconnection precedes hippocampal atrophy in clinically confirmed multi-domain amnestic Mild Cognitive Impairment
Source: EXCLI J. 2021 Sep 27;20:1458–73. doi: 10.17179/excli2021-4191 (PMC8564906; doi:10.17179/excli2021-4191)
Supplement: Supplementary information [file EXCLI-20-1458-s-001.pdf]

EXCLI Journal 2021;20:1458-1473 – ISSN 1611-2156
Received: August 13, 2021, accepted: September 08, 2021, published: September 27, 2021

Supplementary information to:

Original article:

Kathryn M. Broadhouse[1], Natalie J. Winks[2], Mathew J. Summers[3]*

[1] The University of the Sunshine Coast, School of Science and Engineering, Sunshine Coast, QLD, Australia
[2] Sunshine Coast University Hospital, Sunshine Coast Hospital and Health Service, Birtinya, QLD, Australia
[3] The University of the Sunshine Coast, School of Health and Behavioural Sciences, Maroochydore, QLD, Australia

* **Corresponding author:** A/Prof Mathew J. Summers, The University of the Sunshine Coast, School of Health and Behavioural Sciences, Maroochydore, QLD, Australia.
E-mail: msummers@usc.edu.au

http://dx.doi.org/10.17179/excli2021-4191

This is an Open Access article distributed under the terms of the Creative Commons Attribution License (http://creativecommons.org/licenses/by/4.0/).

EXCLI Journal 2021;20:1458-1473 – ISSN 1611-2156
Received: August 13, 2021, accepted: September 08, 2021, published: September 27, 2021

Supplementary File 1

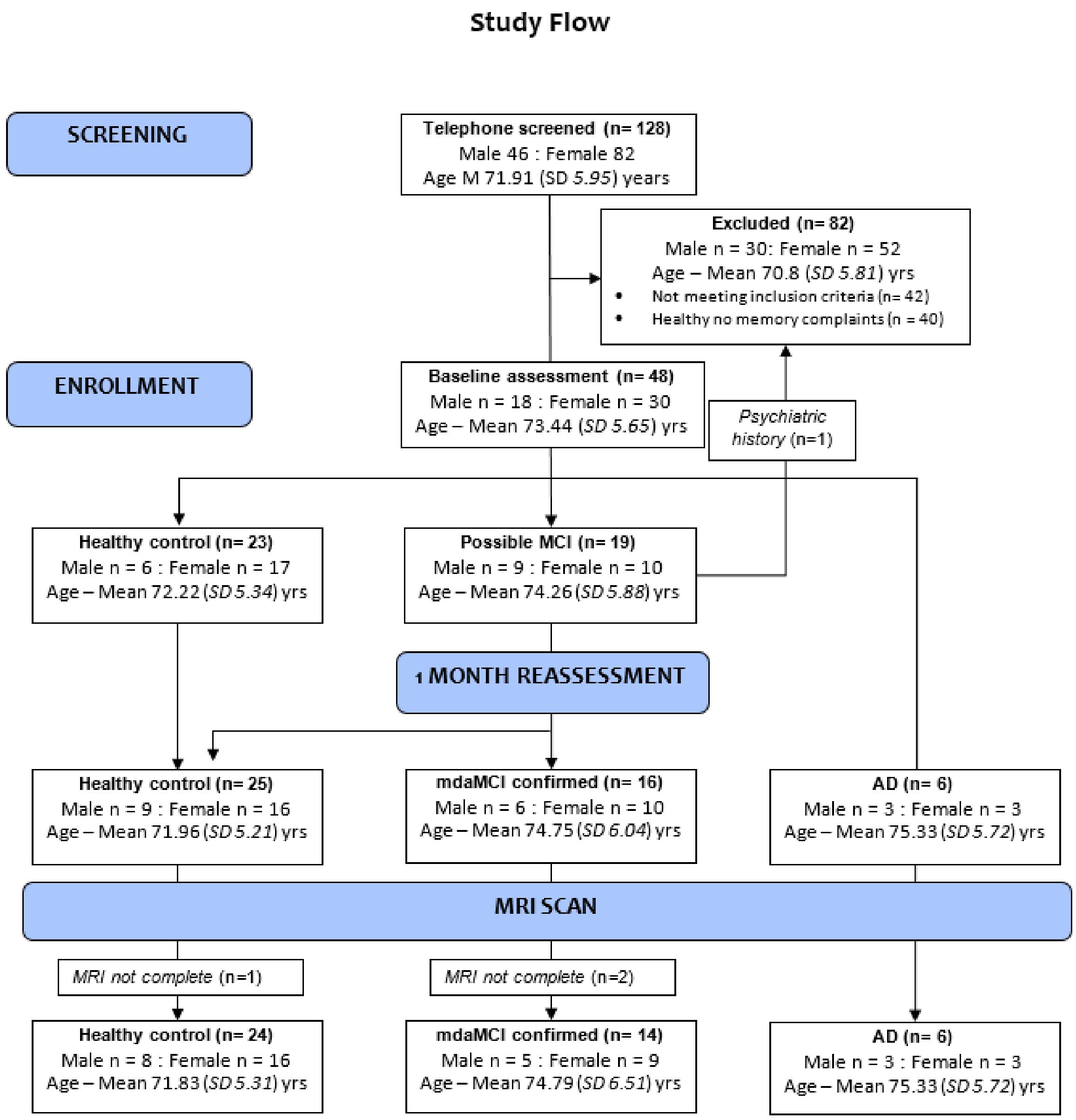

Figure S1: Study flow

EXCLI Journal 2021;20:1458-1473 – ISSN 1611-2156
Received: August 13, 2021, accepted: September 08, 2021, published: September 27, 2021

Supplementary File 2

MRI processing and analysis

Structural analysis: MPRAGE datasets were automatically processed to obtain reliable cortical reconstruction and volumetric segmentations using the *whole-brain* imaging analysis stream in FreeSurfer v6.0. All outputs were visually inspected and where necessary, manual edits were carried out following the FreeSurfer manual editing pipeline. Segmentation of the left and right hippocampi into the head and body subregions were performed with the *hippocampal subfield and nuclei of the amygdala* processing stream FreeSurfer v6.0 (HBT output) using the Freesurfer image analysis suite, which is documented and freely available for download online (http://surfer.nmr.mgh.harvard.edu/).

Functional connectivity analysis: Structural FreeSurfer processed and rsfMRI datasets were pre-processed and analyzed with the open source Matlab/SPM-based CONN toolbox (conn-toolbox.org) following the default DARTEL pre-processing stream. In brief: (i) pre-processing of functional and anatomical volumes using SPM12 realignment, outlier identification, co-registration, segmentation, normalization to standard MNI space (DARTEL stream), smoothing (slice-time was omitted as the multi-slice EPI sequence provided sub 2000 TR). (ii) control of residual physiological and motion artefacts - scrubbing, denoising, global regression, band-pass filtering of 0.01-0.08 Hz and finally regressing out nuisance signals related to white matter, whole-brain and cerebrospinal fluid signal. Individual functional connectivity matrices were then determined using the default FSL Harvard-Oxford Atlas for whole brain ROI-to-ROI analysis.

EXCLI Journal 2021;20:1458-1473 – ISSN 1611-2156
Received: August 13, 2021, accepted: September 08, 2021, published: September 27, 2021

Fronto-temporal-limbic ROI list

```
atlas.FP r (Frontal Pole Right)atlas.FP l (Frontal Pole Left)
atlas.IC r (Insular Cortex Right)
atlas.IC l (Insular Cortex Left)
atlas.SFG r (Superior Frontal Gyrus Right)
atlas.SFG l (Superior Frontal Gyrus Left)
atlas.MidFG r (Middle Frontal Gyrus Right)
atlas.MidFG l (Middle Frontal Gyrus Left)
atlas.IFG tri r (Inferior Frontal Gyrus, pars triangularis Right)
atlas.IFG tri l (Inferior Frontal Gyrus, pars triangularis Left)
atlas.IFG oper r (Inferior Frontal Gyrus, pars opercularis Right)
atlas.IFG oper l (Inferior Frontal Gyrus, pars opercularis Left)
atlas.PreCG r (Precentral Gyrus Right)
atlas.PreCG l (Precentral Gyrus Left)
atlas.TP r (Temporal Pole Right)
atlas.TP l (Temporal Pole Left)
atlas.aSTG r (Superior Temporal Gyrus, anterior division Right)
atlas.aSTG l (Superior Temporal Gyrus, anterior division Left)
atlas.pSTG r (Superior Temporal Gyrus, posterior division Right)
atlas.pSTG l (Superior Temporal Gyrus, posterior division Left)
atlas.aMTG r (Middle Temporal Gyrus, anterior division Right)
atlas.aMTG l (Middle Temporal Gyrus, anterior division Left)
atlas.pMTG r (Middle Temporal Gyrus, posterior division Right)
atlas.pMTG l (Middle Temporal Gyrus, posterior division Left)
atlas.toMTG r (Middle Temporal Gyrus, temporooccipital part Right)
atlas.toMTG l (Middle Temporal Gyrus, temporooccipital part Left)
atlas.aITG r (Inferior Temporal Gyrus, anterior division Right)
atlas.aITG l (Inferior Temporal Gyrus, anterior division Left)
atlas.pITG r (Inferior Temporal Gyrus, posterior division Right)
atlas.pITG l (Inferior Temporal Gyrus, posterior division Left)
atlas.toITG r (Inferior Temporal Gyrus, temporooccipital part Right)
atlas.toITG l (Inferior Temporal Gyrus, temporooccipital part Left)
atlas.MedFC (Frontal Medial Cortex)
atlas.PaCiG r (Paracingulate Gyrus Right)
atlas.PaCiG l (Paracingulate Gyrus Left)
atlas.AC (Cingulate Gyrus, anterior division)
atlas.FOrb r (Frontal Orbital Cortex Right)
atlas.FOrb l (Frontal Orbital Cortex Left)
atlas.aPaHC r (Parahippocampal Gyrus, anterior division Right)
atlas.aPaHC l (Parahippocampal Gyrus, anterior division Left)
atlas.pPaHC r (Parahippocampal Gyrus, posterior division Right)
atlas.pPaHC l (Parahippocampal Gyrus, posterior division Left)
atlas.aTFusC r (Temporal Fusiform Cortex, anterior division Right)
atlas.aTFusC l (Temporal Fusiform Cortex, anterior division Left)
atlas.pTFusC r (Temporal Fusiform Cortex, posterior division Right)
atlas.pTFusC l (Temporal Fusiform Cortex, posterior division Left)
atlas.TOFusC r (Temporal Occipital Fusiform Cortex Right)
atlas.TOFusC l (Temporal Occipital Fusiform Cortex Left)
atlas.FO r (Frontal Operculum Cortex Right)
atlas.FO l (Frontal Operculum Cortex Left)
atlas.Hippocampus r
atlas.Hippocampus l
atlas.Amygdala r
atlas.Amygdala l
atlas.Accumbens r
atlas.Accumbens l
```

EXCLI Journal 2021;20:1458-1473 – ISSN 1611-2156
Received: August 13, 2021, accepted: September 08, 2021, published: September 27, 2021

Supplementary File 3

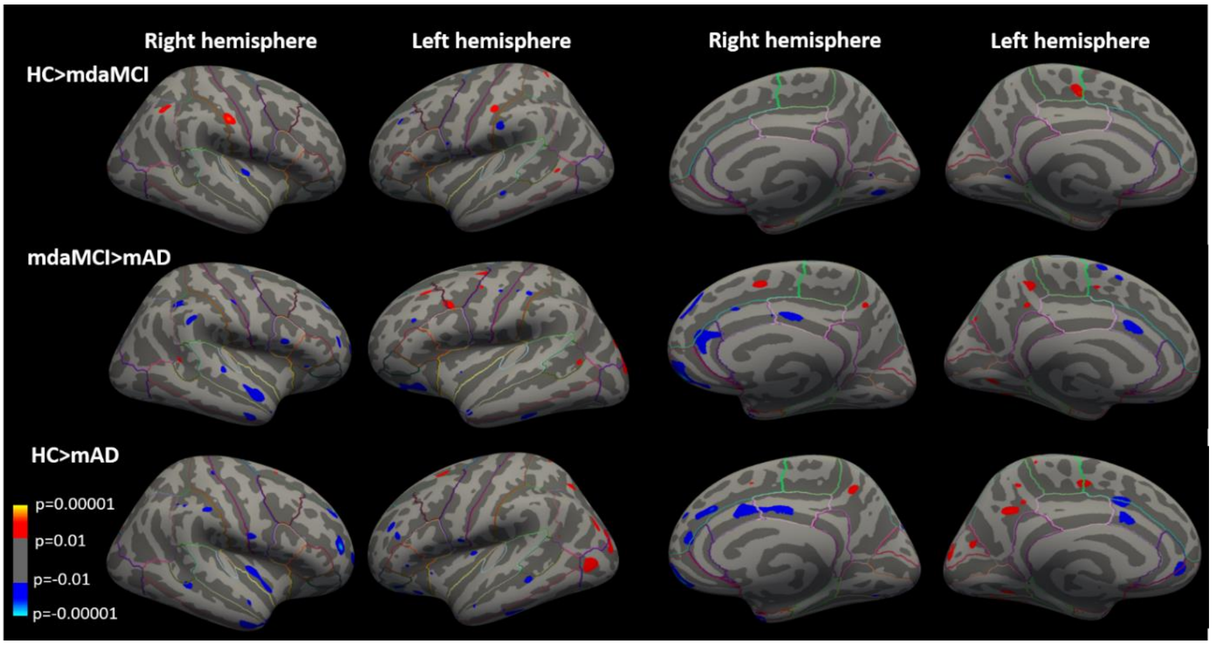

Figure S2: Cortical group analysis. Uncorrected significance maps displaying vertices with p<0.01 for group analysis comparing cortical thickness differences between HC versus MCI (top row), mdaMCI versus mAD (middle row) and HC versus mAD (bottom row) correcting for age. Blue indicates a decrease and red indicates an increase in cortical thickness in group two compared to group one of the tested contrasts. Lateral (1st and 2nd column) and medial (3rd and 4th column) aspects of the left and right hemispheres are shown. Frontal and temporal cortical thinning is observed with disease progression; however, these differences do not survive multiple comparison corrections.
